# The inner critical voice among university students and patients with depression – A linguistic analysis of self-criticism

**DOI:** 10.1371/journal.pone.0337220

**Published:** 2025-12-12

**Authors:** Antonia M. Werner-Riccetti, Ana N. Tibubos, Neele Reiss, Sonja Rohrmann

**Affiliations:** 1 Department of Psychology, Goethe University Frankfurt/Main, Germany; 2 Department of Psychosomatic Medicine and Psychotherapy, University Medical Center of the Johannes Gutenberg University Mainz, Germany; 3 Department of Diagnostics in Healthcare & eHealth, Trier University, Germany; 4 Institute for Psychotherapy Mainz (ipsti-mz), Mainz, Germany; University of Glasgow, UNITED KINGDOM OF GREAT BRITAIN AND NORTHERN IRELAND

## Abstract

Self-criticism is considered an important vulnerability factor for the development and maintenance of psychopathological symptoms. Mostly assessed as trait via self-report, information about its linguistic features is rare. The following study explored individual differences of self-criticism of 184 university students (84.2% female) and 45 inpatients in treatment for a depressive disorder (62.2% female). Participants verbalized self-critical thoughts after a standardized induction of negative emotions, talking out loud to themselves as their “inner critic”. Participants’ self-criticism was analyzed by conducting a linguistic analysis with a quantitative word count tool (Linguistic Inquiry and Word Count – LIWC). Self-criticism was also measured with an established self-report instrument, alongside measures of self-compassion and depressive symptoms for validation. Non-parametric Spearman rank correlations between the linguistic features of the “inner critic”, self-reported self-criticism, self-compassion, and depressive symptoms have been conducted. Furthermore, group differences between university students and patients have been explored. In both subsamples, small significant relations between the language categories and self-reported self-criticism, self-compassion, and depressive symptoms became evident, ranging from |ρ| = .13 to |ρ| = .39. There were slightly different patterns in students and patients, indicating qualitative differences in clinically relevant self-criticism and non-pathogenic self-reflection. University students used significantly fewer pronouns than patients with depression when acting as their “inner critic” (*t* = −5.14, *p* < .001) and less negative emotion words (*t* = −4.19, *p* < .001), particularly words indicating sadness (*t* = −3.18, *p* = .003). Employing different assessment methods of self-criticism and comparing clinical and non-clinical samples adds valuable information for a better understanding of the cognitive-affective processes underlying self-criticism. The results can inform clinical practice and public mental health strategies aimed at promoting mental health and preventing or treating mental disorders.

## Introduction

Excessively harsh self-criticism, i.e., the way individuals judge themselves regarding their behavior, emotions, and thoughts as well as their whole self, is recognized as a transdiagnostic vulnerability factor and a symptom of different mental disorders [1–4]. Most studies on this topic addressed the relation of self-criticism with depressive symptoms [5–8] or eating disorders [9–11], but self-criticism is also related to social anxiety [12,13], suicidality [14], and non-suicidal self-injury [15,16]. For these reasons, self-criticism is something that psychotherapeutic treatments aim to reduce in order to promote a healthy self-to-self relationship [17].

Self-criticism was conceptualized by several clinical psychologists from different theoretical backgrounds, e.g., psychoanalytic [18], psychodynamic [3], and socio-cognitive evolutionary [2,17]. However, as there has not been an integration of these different approaches and models so far [19], our study is based on three concepts of self-criticism, namely the ones of Sidney J. Blatt [18], Golan Shahar [3], and Paul Gilbert [3,17].

We align with Blatt´s view that excessive self-criticism evolves from interruptions in the development of self-definition [18]. Blatt defined self-definition and interpersonal relatedness as the two key dimensions of personality development. The personality dimension of self-definition strives towards being the authentic self, which defines the self mostly in contrast to others, emphasizing a basic need for autonomy and individuality. The other personality dimension, interpersonal relatedness, is expressed by how we experience our interpersonal relationships. The developmental goal would be to build meaningful relationships with others without denying the self, acknowledging that individuals are living in exchange with others and are interdependent.

The two dimensions are intertwined and individuals are in a constant process of integrating both self-definition and interpersonal relatedness. In this dynamic process, self-criticism evolves through disruptions in this integration and appears as the need of achieving very high standards, as only then – if at all – accepting the self is possible. Are those standards not met or mistakes have been made, harsh negative judgement of the whole self, own emotions, cognitions, and behaviors as well as entire self-devaluation may occur. This intra-psychological process is accompanied by feelings of shame and guilt [18] and it does not only affect the self-critical individuals’ mental health in terms of, e.g., depressive symptoms, but also how they interact with others.

Regarding *how* the disruptions in the process of self-definition take place, both, Shahar [3] and Gilbert [2,17], specified that self-criticism develops through the experience of external criticism, e.g., from parents, teachers, or peers [3], and that it can have many different sources of different severity, including early abuse and neglect or bullying [17]. Based on these experiences, individuals who are frequently and/or harshly criticized tend to internalize external criticism and the accompanying feelings of rejection. They also start criticizing themselves without external criticism, for example when failing to achieve an academic goal, in anticipation of a possible rejection by others. This tendency represents a defense mechanism that softens the hurtful experience of being rejected or dismissed.

Gilbert and colleagues further specified self-criticism by defining two different types of self-criticism: self-correction and self-hating [20]. Self-correction is based on criticizing a specific behavior, which is seen as a mistake, whereas self-hating involves a hostile, attacking component and can therefore be regarded as self-attacking [17]. They are related to more (i.e., self-attacking) or less (i.e., self-correction) harmful consequences. The more self-attacking individuals are, the harsher they tend to be on themselves when they make mistakes or fail to meet their high standards regarding their behavior.

In contrast to self-attacking, compassionate self-correction is based on self-compassion. Self-compassion can be defined as a sensitivity for suffering in oneself, the understanding that suffering is universal, and the motivation to alleviate the suffering through sympathizing, empathizing with and taking care for oneself [21]. Even if individuals might have the tendency toward excessive self-criticism, they can learn to correct themselves in a self-compassionate way rather than engage in a hostile and self-attacking form of self-criticism, which may help prevent the development of mental disorders [17,22,23].

Providing biopsychological evidence for the model of two forms of self-criticism, a recent neuropsychological study showed that different physiological markers were activated during hostile self-criticism versus compassionate self-correction [24]. This finding underlines that prevention measures and psychotherapeutic treatments should aim to promote a compassionate form of self-responding, in order to counteract the negative consequences of hostile and excessive self-criticism [25,26].

Based on this conceptualization of self-criticism, *Compassion-Focused Therapy* [CFT], developed by Paul Gilbert [2,27], was found to be effective in a series of meta-analyses for several mental health outcomes, including lowering hostile forms of self-criticism [28]. Moreover, because extreme self-criticism is associated with poorer psychotherapy outcomes [29], addressing self-criticism in mental health promotion and psychotherapy is crucial when treating patients with mental disorders or counseling clients.

In order to gain more knowledge on how self-criticism is expressed verbally, the current study aims to investigate how individual differences in the inner critical voice, i.e., the words individuals use when talking to themselves, are associated with self-reported self-criticism, self-compassion, and depressive symptoms. We think that insights into the concrete language by which individuals address themselves could be utilized to design interventions that promote a more compassionate use of words in communicating with oneself and could help to prevent excessive self-criticism and the development of mental disorders.

### Features of verbalized self-criticism

Until now, most research on self-criticism and its association to mental health has relied on self-report measures. The most commonly used instruments are firstly, the *Depressive Experience Questionnaire* (DEQ) [30] and its shorter versions, such as the 12-item version by Viglione et al. [31] or the ultra-brief screening tool for trait self-criticism DEQ-SC4 [32], all based on Blatt’s work on different forms of depression and the two dimensions of personality summarized in his books [18,33]; and secondly, the *Forms of Self-criticizing/Attacking and Self-Reassuring Scale* (FSCRS) [20], which measures the two forms of self-criticism described by Gilbert [17]. Additionally, nine further, less frequently used self-report measures have been developed for the assessment of self-criticism [34].

Even if the DEQ and FSCRS are well-established and show good psychometric properties, they cannot provide an understanding of whether, for example, the tone of the self-critical voice or the emotions accompanying self-critical thoughts matter for the association between self-criticism and mental health. Addressing this gap, other approaches are needed in order to understand better where mental health interventions should be implemented for change the self-critical process toward desired outcomes.

Only a few studies have used other assessment methods than self-report [35–39]. In their study among college students, Whelton and Greenberg [35] systematically observed the participants’ emotional states by videotaping them while they criticized themselves and responded to this criticism. They found that participants high in self-criticism expressed self-critical thoughts with more contempt and answered more submissively to their critical voice, showing more feelings of sadness and shame compared to controls.

Beside this unique emotional evaluation of expressed self-criticism, Whelton and Henkelmann [36] did a language analysis of verbalized self-criticism regarding its content, using a qualitative approach. They aimed to determine whether less harmful forms of self-criticism also existed and sought to better understand this complex phenomenon. They identified eight categories regarding the verbalized self-criticism, including “demands and orders”, “exhorting and preaching”, “explanations and excuses”, “concern, protection, and support”, “inducing fear and anxiety”, “description”, “explore/puzzle/existential”, “self-attack”, and “condemnation”. They concluded that different forms of self-criticism must be investigated.

More recent studies [37–39] analyzed vocal/acoustic cues as well as facial expressions of participants engaging in a two-chair dialogue, a common psychotherapeutic technique in Emotion-Focused Therapy (EFT) [40–42]. When analyzing vocal cues (i.e., pitch and intensity) of self-criticism, self-protection and self-compassion in 12 EFT two-chair dialogue videos, Bailey and colleagues found that participants expressed self-criticism and self-protection with greater intensity (e. g., louder) compared to self-compassion. Regarding pitch, they reported no significant differences [37]. Analyzing videos of 80 participants criticizing themselves in the two-chair dialogue, Halamová and colleagues observed facial cues indicating contempt, fear, embarrassment, or shame (e.g., Lip Press, Jaw Drop, or Outer Brow Raise) [38]. Additionally, self-protection as possible response to self-criticism was analyzed in terms of vocal cues and facial expressions: Vráblová and colleagues found that highly self-protective participants responded with more contempt and fear, and less joy and surprise, to their self-criticizing compared to participants with low self-protective tendencies [39].

In summary, it appears very useful to explore other ways of assessing and detecting excessive self-criticism through audio- or video-recordings of verbalized self-criticism, in order to enhance our understanding of self-to-self communication. However, none of the mentioned studies analyzed individual differences in the exact language of the verbalized self-criticism, i.e., the words used by the “inner critical voice”. Therefore, we aimed to address this gap in the literature by conducting a linguistic analysis of verbalized self-criticism using a quantitative word-count tool.

### Individual differences in language use

Based on the assumption that the way we talk reflects a part of our personality [43–45], the *Linguistic Inquiry and Word Count (LIWC)* was developed by Pennebaker and colleagues [46,47] with the aim to provide a quantitative way of analyzing language. The LIWC-analysis follows the rationale of counting the words used by an individual and assigning them to a variety of linguistic categories. These categories include standard language categories such as pronouns, but also categories reflecting psychological processes such as positive and negative emotion categories. There are also content-related categories (e.g., occupation-related words) and the tool allows one to create one’s own categories. The categories are assembled in so-called dictionaries, which are available in a variety of languages (e.g., Meier et al., 2018 [48] for German).

LIWC is versatile in its applications to analyzing written data from social media platforms, e.g., *Reddit* [49], online self-presentation [50], or therapeutic settings in the context of written texts, e.g., expressive writing tasks [51,52]. In their most recent study, Mesquiti and colleagues used LIWC-analysis on 45,225,895 *Reddit* comments from 2,451,289 unique users and 30 US cities posted between January 2019 and March 2023. They aimed to understand how collectively experienced social events, e.g., the COVID-19 pandemic or the Black Lives Matter (BLM) protests, were reflected in language [49]. They found changes in the use of anxiety and anger words. For anxiety language shifts, they reported an increased public anxiety following the lockdown due to the COVID-19 pandemic (Cohen’s *d* = 0.25). Regarding anger, they found that the largest shift in anger words for BLM occurred one week after George Floyd’s death (Cohen’s *d* = 0.15). Both findings demonstrate how LIWC-analysis seems a promising approach when utilized in big datasets, to detect a collective emotional response to public issues.

In studies with a clinical focus, Lyons and colleagues analyzed online self-presentation among individuals with anorexia nervosa. They found that pro-anorectic individuals, i.e., persons presenting anorexia nervosa as their lifestyle worth imitating, used more positive emotions and less anxiety language compared to individuals recovering from anorexia [50]. Comparing adolescents with elevated levels of depressive symptoms to healthy controls in a self-evaluation exercise (*N* = 549, aged 13–18), Hards and colleagues found that the adolescents with elevated depressive symptoms used more words classified as negative emotions such as anxiety and sadness words, compared to the healthy ones [51].

In an intervention study, Liehr and colleagues analyzed texts of therapeutic writing tasks to evaluate whether a mindfulness-based intervention for residents in a therapeutic community treating substance use would affect their writing. They found a tendency toward a reduction in the use of negative emotion words in the texts after the intervention [52].

Beyond that, there is fundamental research on associations between word use and other personality traits besides self-criticism such as narcissism or self-acceptance. Holtzman and colleagues analyzed spontaneous language use with LIWC and revealed a small relation (*r* = .10) of grandiose narcissism and more use of words related to sports, second-person pronouns as well as less fear or anxiety words [53]. Looking into the use of pronouns, narcissism shows no correlation with using first-person pronouns, referred to as “I-talk” [54]. However, “I-talk” could be linked to negative emotionality or depressive states [55]. Furthermore, regarding the opposite tendency to self-criticism, which involves accepting one’s own self (trait self-acceptance), Tibubos and colleagues analyzed life narratives of 149 individuals and found that the more self-accepting individuals were, the more emotion words they used [56].

All these studies show individual differences in the use of emotional words, including negative (e.g., anger- or anxiety-related terms) and positive emotion words, either by comparing clinically affected individuals to healthy controls or by examining individuals with varying levels in certain personality traits. However, to our knowledge, there is no study that has analyzed verbalized self-criticism with LIWC and investigated individual differences in word use and their association with self-reported self-criticism, self-compassion, and mental health outcomes.

### Objectives

As the intra-psychological switch to engage in compassionate self-correction instead of hostile self-attacking is a key goal of CFT [17], the aim of the current study is to examine psycho-linguistic features of verbalized self-criticism in order to investigate whether the individual’s inner language reflects extreme levels of self-criticism, and whether it could provide a way to treat excessive self-criticism by shifting the inner dialogue toward a more compassionate and less hostile form of communication based on words choice.

Building upon the work on self-criticism described above [35–39], the first aim of our study was to describe the linguistic features of verbally expressed self-criticism using LIWC-2015 [46,47], focusing on total word count, use of pronouns, and use of emotional words (positive and negative). University students without a mental disorder were compared to patients in treatment for a depressive disorder. We expected that patients would differ in the way they verbalized self-criticism, with their “inner critic” expressing more words overall, using more negative emotion words, and the singulat pronoun “you” compared to a healthy control group of university students.

In the next step, we tested associations of the words used by the individual’s inner critical voice, representing self-criticism in a specific situation (state self-criticism), and the relatively stable disposition to engage in self-criticism (trait self-criticism) measured via self-report. This was done to distinguish between the relatively stable trait self-criticism and temporary states of self-criticism [57]. Testing for associations between word use in state self-criticism and self-reported trait self-criticism contributes to a more complementary assessment of self-criticism beyond self-report.

We expected individuals who described themselves more self-critical in the self-report would produce a higher number of words when assuming the role of their inner critical voice and would use negative emotion words and pronouns more frequently than individuals with lower levels of trait self-criticism. As we recorded the embodied “inner critic” and instructed participants to address themselves using the pronoun “you”, we expected that greater use of “you” would indicate higher levels of self-criticism.

Furthermore, we explored associations of the linguistic features of verbalized self-criticism with self-compassion, the positive counterpart of hostile self-criticism, as well as with (self-reported) depressive symptoms, expecting negative correlations with self-compassion and positive correlations with depressive symptoms. Here, we also explored whether these correlational patterns differed between a clinical sample of patients with depression and a non-clinical sample of university students.

## Materials and methods

### Participants

Overall, *N* = 261 participants took part in the study, one non-clinical subsample of university students (*n* = 206 participants, 84.5% female), and one clinical subsample of patients, who were all inpatients in a medical facility at the time of their participation (*n* = 55, 63.6% female). For the current analyses, *n* = 16 students had been excluded to avoid the risk of potential bias in the non-clinical subsample, as they reported to be currently in psychotherapeutic treatment. Furthermore, six additional students were excluded: one for having an age below 18 years, five for having no transcripts of their verbalized self-criticism due to technical reasons (*n* = 4) or not saying anything (*n* = 1). Therefore, finally *n* = 184 students (84.2% female) between 18 and 48 years (*M* = 23.2 years, *SD* = 5.4 years) were included in the current analyses. The education level among the students was naturally homogeneous with the majority (85%, *n* = 156) reporting a high school diploma, 14.7% (*n* = 27) holding a bachelor’s or other university degree, and one participant (0.5%) having a professional school degree.

Patients with depression in the clinical subsample were all currently inpatients at the cooperating psychiatric clinic. We excluded patients who also had a diagnosis of a borderline personality disorder (BPD, *n* = 7) as well as patients with missing details on their diagnosis, as we could not be sure they met the inclusion criterion of having no comorbidity to the depressive disorder (*n* = 3). Finally, data of *n* = 45 patients (62.2% female) were analyzed in this study. The patients were aged between 18 and 74 years with an average age of *M* = 42.0 years (*SD* = 15.9). The patient sample depicted the whole range of educational levels from elementary school degree to university graduation. There were significant differences between the two subsamples regarding age, marital status, and education level. Table 1 summarizes the socio-demographic information of both subsamples.

**Table 1:** Socio-demographic and psychological characteristics of the total study sample as well as of the two separately analyzed subsamples (university students and patients with depression).

	Total sample (*n* = 229)	Students (*n* = 184)	Patients with Depression (*n* = 45)	*p*-value
Gender, *N* (%)				.06, n.s.^1^
Male	46 (20.1)	29 (15.8)	17 (37.8)	
Female	183 (79.9)	155 (84.2)	28 (62.2)	
Age				
Age in years, *M* (*SD*)	27.0 (11.4)	23.2 (5.4)	42.0 (15.9)	<.001^2^
Age range in years	18-74	18-28	18-74	
Missing, *N* (%)	3 (1.3)	3 (1.6)	0 (0.0)	
Marital status				
Single, *N* (%)	196 (85.6)	173 (94.0)	23 (51.1)	<.001^1^
Married, *N* (%)	19 (8.3)	6 (3.3)	13 (28.9)	
Living separated, *N* (%)	3 (1.3)	0 (0.0)	3 (6.7)	
Divorced, *N* (%)	7 (3.1)	3 (1.6)	4 (8.9)	
Bereaved, *N* (%)	2 (0.9)	0 (0.0)	2 (4.4)	
Missing, *N* (%)	2 (0.9)	2 (1.1)	0 (0.0)	
Education				
College or university degree, *N* (%)	30 (13.1)	27 (14.7)	3 (6.7)	<.001^1^
Professional school degree, *N* (%)	5 (2.2)	1 (0.5)	4 (8.9)	
High school diploma, *N* (%)	165 (72.1)	156 (84.8)	9 (20.0)	
Secondary school of ten years, *N* (%)	21 (9.2)	0 (0.0)	21 (46.7)	
Secondary school of nine years, *N* (%)	6 (2.6)	0 (0.0)	6 (13.3)	
Elementary school, *N* (%)	2 (0.9)	0 (0.0)	2 (4.4)	

*Note.* ^1^Kolmogorov Smirnov test; ^2^*t*-test

### Procedure

The data collection took place between August 3, 2015, and May 13, 2017. It consisted of two separate parts: an online survey and a lab session, the latter following an experimental approach.

### Online survey

The student sample was recruited through online newsletters and flyer information at various universities in the Rhine-Main area, Germany. Interested individuals received a link to an online survey, which included, among other measures, assessments of self-compassion, depressive symptoms, and socio-demographic questions such as age, gender, marital status, educational level, field of study, and semester number, as well as health-related questions (e.g., whether they had a diagnosis of a physical or mental disorder, were currently receiving treatment for a mental disorder, or regularly took medication). Participants were instructed to complete the questionnaire thoroughly at home and were informed that, upon finishing the survey, they would receive a lab appointment for the second part of the study.

In the patients’ sample, the procedure was slightly different. All patients were inpatients at the cooperating medical center for psychiatry and psychotherapy and were informed about the study by the collaborating physician. The physician was instructed to approach only patients with adequate emotional stability. The diagnoses of participating patients were extracted from their medical files by the cooperating physician, who communicated them to the study team while the team was on site for the sessions during the data collection phase.

Patients who agreed to participate in the study answered the online survey directly in the medical center, with a study assistant present to answer questions or provide assistance in case of any problems with the survey. The questionnaire was the same as that used for the student sample, except for the socio-demographic section: patients were not asked about their field of study, semester number, or current participation in psychotherapeutic treatment. Instead, they reported their employment status, previous therapy experience, and the duration of their inpatient stay at the medical center. Once a participant completed the online survey, the study assistant and the patient scheduled a second appointment for the next part of the study, which also took place at the medical center.

### Lab session

The second part of the study met the criteria of a quasi-experimental design, following the same standardized procedure in the student sample and the sample of patients with depression. A student lab assistant as well as a trained psychologist conducted the experiment together. At first, the lab assistant instructed the participant to answer some questionnaires (including a self-report measure for self-criticism). Then a success-failure manipulation (SFM) took place in order to induce a negative emotional state.

According to a meta-analysis by Nummenmaa and Niemi [58], SFM is effective to induce specific emotional states in experimental settings with high ecological validity, especially when the presented task is of relevance for the participants. In case of the current study, we asked the participants to complete a verbal intelligence performance test. To do so, the lab assistant presented a very difficult anagram task (words whose letters had been rearranged and the task was to find the original word) on a laptop, which the participants were instructed to solve. To induce the experience of failing or underperforming, the anagrams were very long and were presented only for a short time (10 seconds), making the task hardly solvable except for a few easier items, and some were unsolvable altogether. At the end of the test, participants received a fake feedback on their screen indicating that they had solved only 17% of the presented anagrams and were therefore below average compared to their reference group. Finally, the lab assistant asked the participants for their results and responded: “Oh, okay? So not so good.”. This procedure was intended to ensure the perception of underperformance in the task. Afterward, the lab assistant left the room, and the psychologist, trained for the next step of the study, entered.

The psychologist used the two-chair dialogue [40–42], primarily practiced in EFT, to evoke the participants' self-critical voice. In this technique, participants are asked to embody a specific part of their self, for example, in our case, the “inner critic”, by expressing it to an empty chair positioned in front of them. Afterwards, participants are usually asked to move to the previously empty chair and respond as another part of the self or the whole self to the chair they previously sat on.

In our study, the psychologist introduced this part by explaining that there are different parts of the self, including a self-critical part called the “inner critic”. The participants were then asked to move to another chair and embody their “inner critic” by verbally criticizing themselves for three minutes, addressing the now empty chair where they had previously sat and completed the anagram task. This verbalized self-criticism was recorded on an audio device. While recording, the psychologist followed a scripted protocol and only asked “What else?” or “What else does the inner critic say?”. After three minutes, the participants were asked to indicate how stressed they felt at that moment on a visual scale ranging from 0 to 100, as well as to answer additional questions about their current emotional state. Each participant who reported a perceived stress value of 50 or higher took part in one of four short intervention conditions (each lasting three minutes) designed to regulate their emotions. The four conditions were: distraction (coloring a mandala), expressive writing (writing down their thoughts and feelings), responding to their critical voice from a self-confident perspective (the second part of the two-chair-dialogue guided by the psychologist); and a control condition, in which participants were instructed to simply wait on the chair and look out of the window for three minutes. Participants who reported a value below 50 had a cooling down phase of three minutes. Every participant was debriefed about the manipulated anagram task afterwards, and that the study was about how they dealt emotionally with the experience of failure, in this case failing in the anagram task.

After each lab session, the audio data were transferred on a computer and transcribed. Once transcribed, the audios have been deleted. The transcripts represent the text data, which have been analyzed regarding their linguistic features.

### Ethical statement

Before participating in the study, all participants of both samples received written informed consent outlining the study procedure, data collection, and anonymization. They were informed that they could withdraw from the study at any time without any consequences. A full debriefing was provided after the second part of the study, explaining the manipulated anagram task in the quasi-experimental setting and providing contact information in case of acute emotional distress. The patients’ therapists and nurses were informed about the study and were available to assist in the event of a psychological crisis.

The study has been carried out in accordance with *The Code of Ethics of the World Medical Association (Declaration of Helsinki)* for experiments involving humans and the *Ethical Principles and Guidelines for the Protection of Human Subjects of Research* of the American Psychological Association (APA). The study was ethically approved by the responsible ethical commission of the university’s Department of Psychology (no. 2014−121) on July 2, 2015. In both subsamples, there was no major incentive to participate: university students and patients each received a small token at the end of the lab session. Additionally, psychology students could earn credit points (*Versuchspersonenminuten*, “VPM”), which are mandatory in their curriculum. While the number of VPM that students could earn through participation was communicated in advance, it was not disclosed during recruitment that participants would also receive a small token.

### Measures

The linguistic analysis of the verbalized self-criticism data was conducted using the computer-based word count program LIWC-2015 [46,47]. The analysis employed the most recent version of the German LIWC dictionary available at the time, which had been validated for its psychometric properties [48]. As relevant categories, number of words (LIWC_num), use of pronouns (LIWC_pronouns-overall, LIWC_personal-pronouns, LIWC_you-total, LIWC_you-singular), LIWC-affect (LIWC_affect-overall), LIWC-positive emotions (LIWC_posEm), LIWC-negative emotions (LIWC_negEm), as well as LIWC-anger (LIWC_ang), LIWC-anxiety (LIWC_anx), and LIWC-sadness (LIWC_sad) were examined.

LIWC counts all the words of one transcript and assigns them to the chosen categories of the activated dictionary. As output, LIWC presents the category *LIWC-num* as total number of words. Regarding all the other categories, the percentage of assigned words per category in relation to the total number of words is presented. A word can be assigned to more than one category.

Self-criticism was assessed with self-report measure *Theoretical Depressive Experiences Questionnaire – 12* (TDEQ-12) [31] in the German version [59]. It consists of 12 items, seven of which asking for self-critical thoughts such as „I have a difficult time accepting weaknesses in myself.“. Participants answer on a 7-point Likert scale ranging from 1 = „strongly disagree“to 7 = „strongly agree“. High values indicate high self-criticism. Internal consistency of the scale of self-criticism in the actual sample was ω = .80 in the student sample (*n *= 179), and ω = .77 in the patient sample (*n *= 43).

Depressive symptoms have been assessed by presenting the *Beck Depression Inventory II* (BDI-II) [60]. The BDI-II assesses depressive symptoms with overall 21 items. Each item represents one depressive symptom, which participants answer on a scale, ranging from 0 to 3, measuring severity of each symptom with 0 = “not present” to 3 = “strongly present” with phrasing fitting to each item. Internal consistency was ω = .87 in the students’ sample (*n* = 184) and ω = .91 in the patients’ sample (*n* = 45).

To measure self-compassion, we used the *Self-Compassion Scale* (SCS) developed by Kristin D. Neff [61]. The SCS consists of 26 items which are attained to six subscales: *Self-Kindness*, *Common Humanity*, and *Mindfulness* representing a positive second-order factor, referred to as *Self-compassionate responding* (SCS_pos) in the following; and *Self-Judgement*, *Isolation*, and *Over-Identification* building another second-order-factor, *Self-uncompassionate responding* (SCS_neg) as proposed by some authors [62,63]. Participants answer to each item on a five-point Likert scale ranging from 1 = ”almost never” to 5 = ”almost always”. A total score can be built by inverting the items of *Self-Judgement*, *Isolation*, and *Over-Identification* and then summing up all 26 items (SCS_total). In the current study, we only used the clearly positive factor SCS_pos in the analyses. Internal consistency (McDonald’s Omega) in the total sample was excellent with ω = .91 for SCS_pos (*n* = 222). Looking at the two subsamples separately, internal consistency was ω = .87 in the students’ sample (*n* = 177), and ω = .89 in the patients’ sample (*n* = 45).

### Data preparation

For the LIWC-analysis, the transcripts were spell-checked and standardized regarding the spelling of filler words such as “eh”. In addition, everything that the psychologist has said was taken out of the transcript. Overall, a total number of 229 transcripts were prepared to analyze. Regarding the self-report measures, we firstly conducted a missing data analysis, which revealed that each variable had between 0 and 7 missing values, corresponding to 0–1.3% missing data per variable. We decided to impute these data for items of the TDEQ-12, the SCS_pos, and the BDI-II with a simple imputation method. We then conducted the scoring procedures for each scale resulting in sum scores for TDEQ-SC, SCS_pos, and BDI-II.

### Statistical analysis

We calculated frequencies for categorical variables (gender, marital status, educational background) as well as means and standard deviations for continuous variables (age, LIWC-categories, BDI-II, TDEQ-SC, and SCS_pos) in the total sample and separately for the two subsamples of university students and patients. In this context, we also looked for outliers in the data and tested each variable for normality. Testing for differences between the clinical and the non-clinical subsample, we compared means for each LIWC-category with *t*-tests. For the *t*-tests, we defined a Bonferroni-corrected α level of α < 0.005 as statistically significant.

To investigate whether linguistic features of verbalized self-criticism are associated with self-reported self-criticism, self-compassion, and depressive symptoms, we used Spearman rank correlations in the total sample as well as separately for each subsample, interpreting an α level of α < .05 as significant.

Finally, we conducted forward stepwise regression analyses with either TDEQ-SC, SCS_pos, or BDI-II as outcome variable. In the first step, we included age (continuous) and subsample (0 = students; 1 = patients) as predictor variables (Model 1). In the second step, we added the LIWC-categories number of words (LIWC_num), you-singular (LIWC_you-singular), affect (LIWC_affect), positive emotions (LIWC_posEm), negative emotions (LIWC_negEm), anxiety (LIWC_anx), anger (LIWC_ang), and sadness (LIWC_sad; all continuous variables) into the regressions (Model 2). Here, we also applied an α level of α < .05 for statistical significance. Figures were developed using RStudio (R version 4.3.3), particularly with the packages *ggplot2* [64] and *hrbrthemes* [65].

## Results

A summary of the descriptive statistics for the variables trait self-criticism, self-compassion, and depressive symptoms is presented in Table 2, including a comparison of the two subsamples. Patients scored significantly higher on self-criticism, as measured by the TDEQ-SC, and had higher BDI-II scores compared to students. Conversely, with regard to self-compassion, students reported higher SCS_pos scores than patients.

**Table 2:** Descriptive statistics of self-criticism, self-compassion, and depressive symptoms in all samples.

	Total sample (*n* = 229)	Students (*n* = 184)	Patients (*n* = 45)	
Study Variable	*M* (*SD*)	*M* (*SD*)	*M* (*SD*)	*p*-value
Self-criticism (TDEQ-SC)	30.90 (7.59)	29.35 (7.00)	37.24 (6.60)	<.001^2^
Self-compassion (SCS_pos)	38.52 (10.07)	40.97 (8.53)	28.51 (9.77)	<.001^2^
Depressive Symptoms (BDI-II)	12.51 (10.85)	8.89 (7.08)	27.31 (11.09)	<.001^2^

*Note.* Reported descriptive statistics are mean value (standard deviation); ^1^Kolmogorov Smirnov test; ^2^*t*-test; TDEQ-SC: Theoretic Depressive Experiences Questionnaire – Self-criticism subscale; SCS_pos: Self-Compassion Scale – self-compassionate responding subscale; BDI-II: Beck’s Depression Inventory-II.

**Fig 1:**
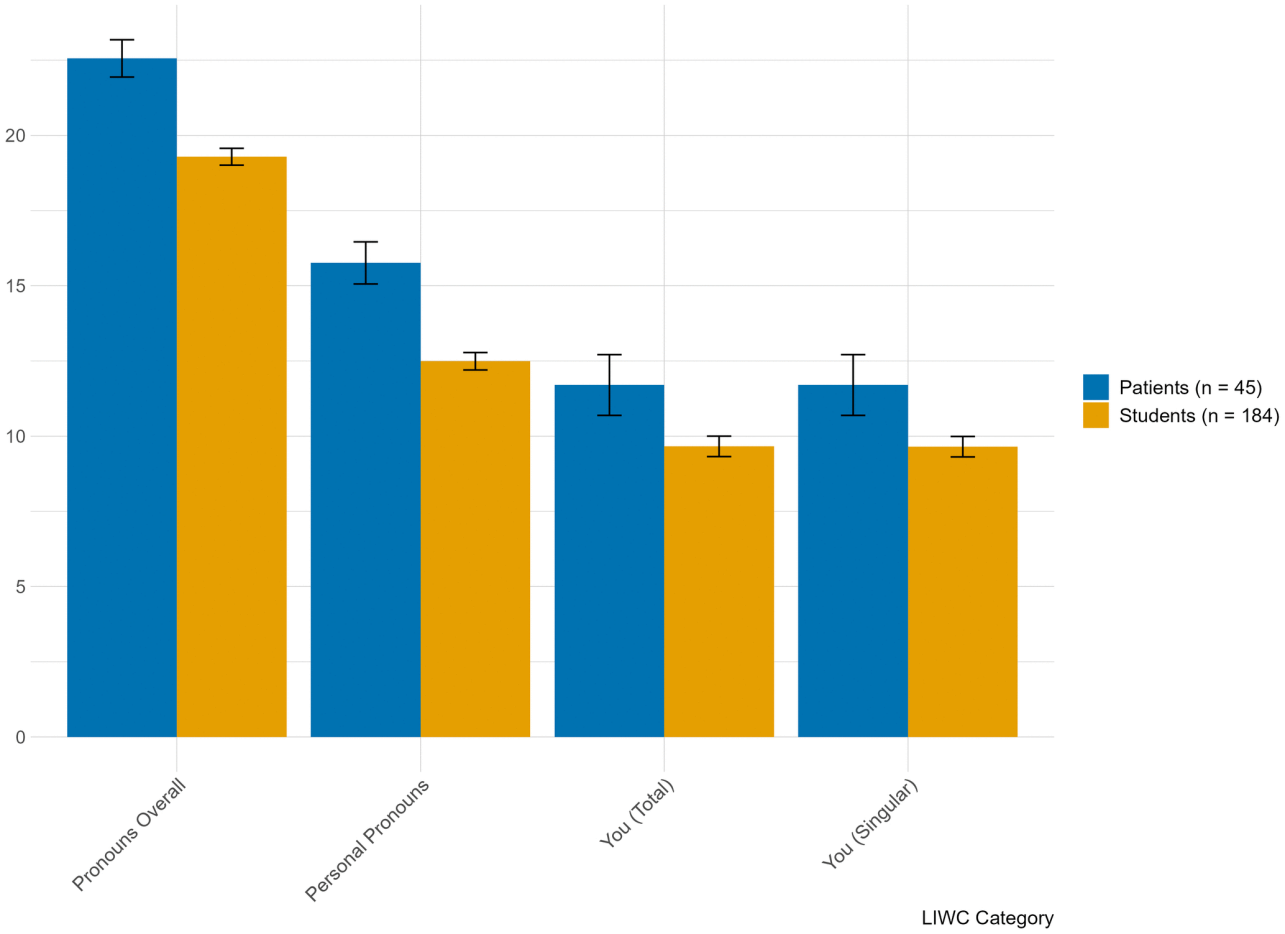
Mean percentage use of pronouns in verbalized self-criticism among university students and patients in treatment for depression.

**Fig 2:**
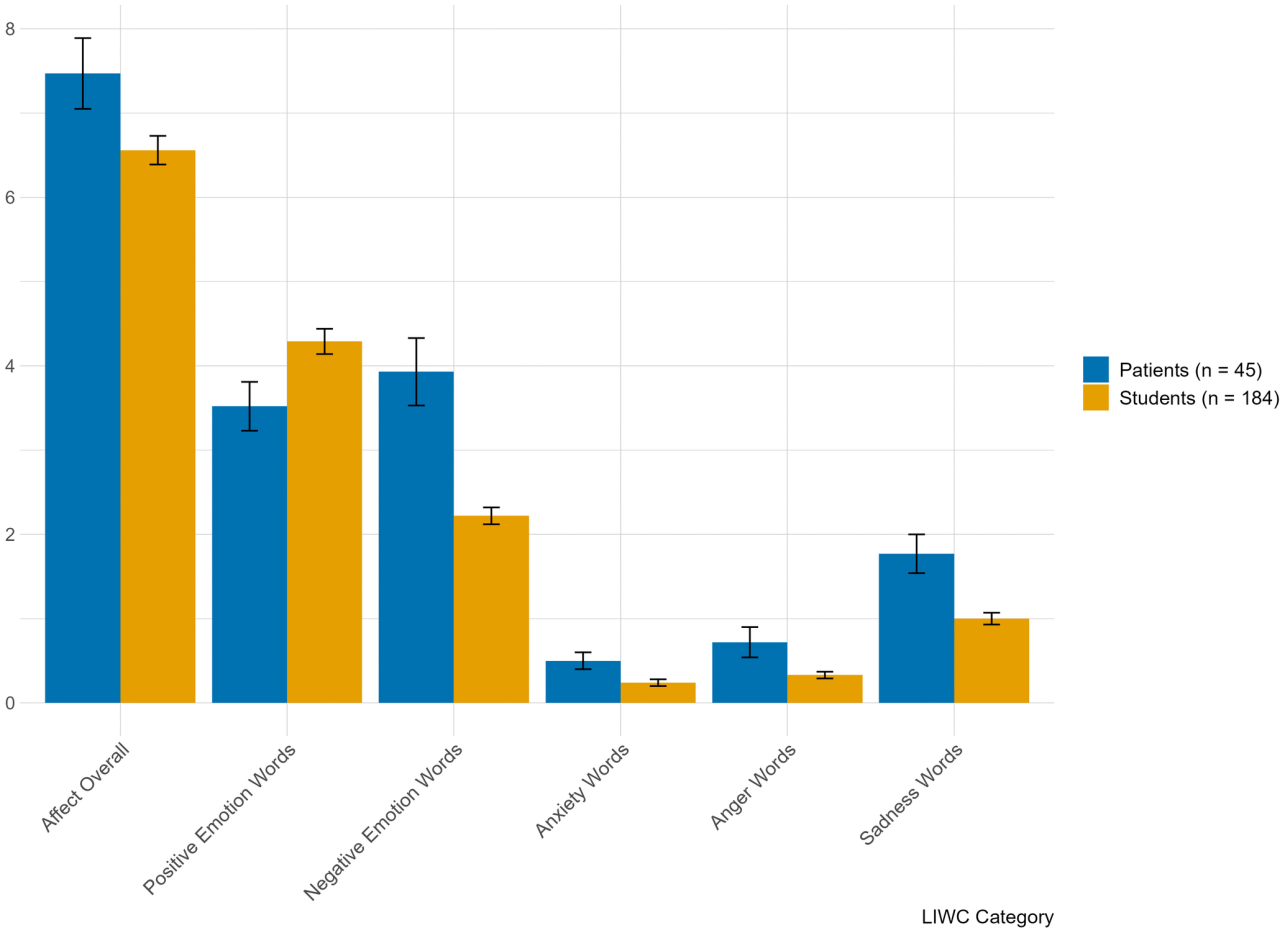
Mean percentage use of emotional words in verbalized self-criticism among university students and patients in treatment for depression.

Fig 1 and Fig 2 present the descriptive statistics (means and standard deviations) per LIWC-category and their differences between the samples. Comparing the patients’ and the students’ sample, the *t*-tests revealed that students used more words in total (*t* = 3.85; *p* < .001; *d* = .64), but less pronouns (overall and personal pronouns, *t* = −5.14 and *t* = −4.84, respectively, with both *p* < .001, *d* = −.52 and *d* = −.41) as well as less negative emotion words, especially less words of sadness (*t* = −4.19, *p* < .001, *d* = −.70; and *t* = −3.18, *p* = .003, *d* = −.39, respectively), compared to patients with depression. These results are all representing medium-sized effects. There was also a medium to large effect regarding positive emotion words, which students used comparably more that patients (*t* = 2.29; *p* = .023; *d* = .71). We observed small effects on use of anxiety and anger words (*t* = −2.43; *p* = .018; *d* = −.16 and *t* = −2.10; *p* = .041; *d* = −.22, respectively), which patients both used more in their verbalized self-criticism.

### Associations of linguistic features of verbalized self-criticism with trait self-criticism, self-compassion, and depressive symptoms

Significant correlations of the LIWC-categories with self-reported self-criticism (TDEQ-SC), self-compassionate responding (SCS_pos), and depressive symptoms (BDI-II) ranged between |ρ| = .13 und |ρ| = .27, representing small effects. Looking at the correlations of LIWC-categories with self-reported self-criticism (TDEQ-SC scores), the strongest positive association became evident between TDEQ-SC scores and the use of the pronoun “you” (ρ = .24; *p* < .001), indicating the use of you mirroring higher self-reported self-criticism. Conversely, participants, who used more positive emotion words in their self-critical voice, had lower scores in the TDEQ-SC (ρ = −.24; *p* = .001). More in detail even, it became evident, the more words of anxiety and anger the inner critical voice used, the higher were the self-reported self-criticism scores (ρ = .15; *p* = .024; ρ = .14; *p* = .037, respectively).

Some differences of the correlational patterns occurred between the students’ and the patients’ sample. Only in students, there was a relation of the use of any type of emotion words from the inner critical voice and lower self-reported self-criticism (ρ = −.19; *p* = .009). The more emotion words were used, the lower were the TDEQ-SC scores. Furthermore, only in patients was a significant positive correlation of the inner critic using negative emotion words and higher TDEQ-SC scores (ρ = .33; *p* = .027). See Table 3 for an overview of all Spearman rank correlations between the LIWC-categories and TDEQ-SC scores.

**Table 3:** Spearman rank correlations (Rho) between linguistic features of verbalized self-criticism and self-reported self-criticism (TDEQ_SC) for the total study sample (*N* = 229), and separately for both subsamples of university students (*n* = 184) and patients with depression (*n* = 45).

	TDEQ_SC		
	total	students	patients
LIWC_num	−.01	.05	.22
LIWC_you-singular	.24**	.21**	.22
LIWC_affect-overall	.08	−.19**	−.01
LIWC_posEm	−.24**	−.22**	−.13
LIWC_negEm	.13	−.07	.33*
LIWC_anx	.15*	.03	.25
LIWC_ang	.14*	.08	.23
LIWC_sad	.05	−.08	.20

*Note.* LIWC-number of words (LIWC_num), use of the pronoun “you” (LIWC_you-singular), LIWC-affect (LIWC_affect-overall), LIWC-positive emotions (LIWC_posEm), LIWC-negative emotions (LIWC_negEm), as well as LIWC-anger (LIWC_ang), LIWC-anxiety (LIWC_anx), and LIWC-sadness (LIWC_sad). *p < .05. ** *p* < .01.

Regarding self-compassion (SCS_pos scores), in the total sample the strongest association was observed between SCS_pos scores and negative emotion words (ρ = −.27; *p* < .001). No significant correlations were found between the inner critical voice with self-compassion in the student sample. Only in patients higher scores in self-compassionate responding correlate with lower use of the pronoun “you” (ρ = −.33; *p* = .027) and with an overall lower word count (ρ = −.39; *p* = .008). Correlations between all tested LIWC-categories and SCS_pos scores are presented in Table 4.

**Table 4:** Spearman rank correlations (Rho) between linguistic features of verbalized self-criticism and self-compassion (SCS_pos) for the total study sample (*N* = 229), and separately for both subsamples of university students (*n* = 184) and patients with depression (*n* = 45).

	SCS_pos		
	total	students	patients
LIWC_num	.08	.03	−.39**
LIWC_you-singular	−.12	.01	−.33*
LIWC_affect-overall	−.09	−.04	.07
LIWC_posEm	.12	.03	−.03
LIWC_negEm	−.27**	−.09	−.19
LIWC_anx	−.10	.03	−.12
LIWC_ang	−.17**	−.11	−.25
LIWC_sad	−.16*	−.03	−.16

*Note.* LIWC-number of words (LIWC_num), use of the pronoun “you” (LIWC_you-singular), LIWC-affect (LIWC_affect-overall), LIWC-positive emotions (LIWC_posEm), LIWC-negative emotions (LIWC_negEm), as well as LIWC-anger (LIWC_ang), LIWC-anxiety (LIWC_anx), and LIWC-sadness (LIWC_sad). *p < .05. ** *p* < .01.

Examining correlations between BDI-II scores and the linguistic features of the inner critical voice, the strongest association in the total sample was observed with positive emotion words (ρ = −.21; *p* = .002), such that fewer positive emotion words in the inner critical voice were associated with higher the BDI-II scores. The total number of words used by the inner critical voice was associated with higher BDI-II scores in students (ρ = .26; *p* < .001) as well as in patients (ρ = .33; *p* = .029). Beyond that, only in the patients’ sample, more negative emotion words were associated with higher BDI-II scores (ρ = .38; *p* = .011). An overview for all correlations between LIWC-categories and BDI-II scores is presented in Table 5.

**Table 5:** Spearman rank correlations (Rho) between linguistic features of verbalized self-criticism and depressive symptoms (BDI-II) for the total study sample (*N* = 229), and separately for both subsamples of university students (*n* = 184) and patients with depression (*n* = 45).

	BDI-II		
	total	students	patients
LIWC_num	.06	.26**	.33*
LIWC_you-singular	.13*	.06	.15
LIWC_affect-overall	−.04	−.18*	.11
LIWC_posEm	−.21**	−.16*	.01
LIWC_negEm	.13*	−.10	.38*
LIWC_anx	.09	−.04	.07
LIWC_ang	.05	−.02	.09
LIWC_sad	.11	−.03	.29

*Note.* LIWC-number of words (LIWC_num), use of the pronoun “you” (LIWC_you-singular), LIWC-affect (LIWC_affect-overall), LIWC-positive emotions (LIWC_posEm), LIWC-negative emotions (LIWC_negEm), as well as LIWC-anger (LIWC_ang), LIWC-anxiety (LIWC_anx), and LIWC-sadness (LIWC_sad). *p < .05. ** *p* < .01.

We also conducted several regression analyses to examine whether the LIWC categories explained variance in self-reported self-criticism, self-compassion, and depressive symptoms beyond age and subsample (students vs. patients). A summary of all regression results is presented in Table 6.

**Table 6:** Forward step-wise linear regressions on self-reported self-criticism, self-compassion, and depressive symptoms with age and subsample (Model 1), as well as additionally with the LIWC-categories number of words (LIWC_num), use of the pronoun “you” (LIWC_you-singular), use of emotion words (LIWC_affect-overall), use of positive emotion words (LIWC_posEm), use of negative emotion words (LIWC_negEm), anxiety (LIWC_anx), anger (LIWC_ang), and sadness (LIWC_sad) in Model 2.

	Self-criticism (TDEQ-SC)						Self-compassion (SCS_pos)						Depressive symptoms (BDI-II)					
	Model 1 (R^2^ = .17***)						Model 1 (R^2^ = .24***)						Model 1 (R^2^ = .45***)					
	*B*	*SE*	95% CI L	95% CI U	*t*	*p*	*B*	*SE*	95% CI L	95% CI U	*t*	*p*	*B*	*SE*	95% CI L	95% CI U	*t*	*p*
Age	−0.08	0.05	−0.18	0.03	−1.40	.16	0.05	0.07	−0.09	0.18	0.70	.48	0.00	0.06	−0.12	0.13	0.04	.97
Subsample	9.23	1.53	6.21	12.26	6.02	**<.001**	−13.30	1.96	−17.16	−9.45	−6.80	**<.001**	18.27	1.78	14.75	21.79	10.24	**<.001**
	**Self-criticism (TDEQ-SC)**						**Self-compassion (SCS-pos)**						**Depressive symptoms (BDI-II)**					
	Model 2 (R^2^ = .20***)						Model 2 (R^2^ = .25***)						Model 2 (R^2^ = .48***)					
	*B*	*SE*	95% CI L	95% CI U	*t*	*p*	*B*	*SE*	95% CI L	95% CI U	*t*	*p*	*B*	*SE*	95% CI L	95% CI U	*t*	*p*
Age	−0.05	0.06	−0.16	0.06	−0.91	.36	0.03	0.07	−0.11	0.17	0.46	.65	0.03	0.06	−0.10	0.16	0.48	0.63
Subsample	8.57	1.58	5.44	11.69	5.41	**<.001**	−12.25	2.05	−16.29	−8.20	−5.97	**<.001**	0.67	1.83	14.60	21.81	9.95	**<.001**
LIWC_num	0.01	0.01	0.00	0.02	1.47	.14	−0.01	0.01	−0.02	0.00	−1.42	.16	0.22	0.01	0.01	0.03	3.98	**<.001**
LIWC_you-singular	0.26	0.10	0.07	0.45	2.72	**.01**	−0.14	0.12	−0.38	0.11	−1.10	.27	0.10	0.11	0.00	0.44	2.00	**.05**
LIWC_affect-overall	2.68	2.71	−2.66	8.03	0.99	.32	−5.16	3.51	−12.08	1.76	−1.47	.14	−0.22	3.13	−7.16	5.19	−0.32	.75
LIWC_posEm	−3.04	2.70	−8.36	2.27	−1.13	.26	5.08	3.49	−1.81	11.96	1.45	.15	0.16	3.11	−5.29	6.99	0.27	.79
LIWC_negEm	−2.80	2.79	−8.30	2.69	−1.01	.32	4.19	3.61	−2.93	11.31	1.16	.25	0.25	3.22	−4.84	7.85	0.47	.64
LIWC_anx	0.58	1.08	−1.55	2.70	0.53	.59	0.65	1.40	−2.10	3.41	0.47	.64	−0.05	1.25	−3.53	1.39	−0.86	.39

First, we conducted three regression analyses, one for each outcome variable, including only age and subsample as predictor variables (Model 1). All models were significant, explaining 17, 24, and 45% of the variance for TDEQ-SC scores, SCS_pos scores, and BDI-II scores, respectively. In all three regressions, a statistically significant association was found with the predictor variable “Subsample”. Specifically, being in the clinical subsample of patients was associated with higher scores on the TDEQ-SC and the BDI-II (*B* = 9.23, *p* < .001; *B* = 18.27, *p* < .001, respectively), as well as lower scores on the SCS_pos (*B* = −13.30, *p* < .001), compared to the non-clinical sample of university students.

In the second step, we included also the LIWC-categories as predictor variables (Model 2). Again, all three models were significant and explained 20, 25, and 48% of the variance in TDEQ-SC scores, SCS_pos scores, and BDI-II scores, respectively, with a significant change in *R²* for TDEQ-SC and BDI-II, but not in SCS_pos. “Subsample” remained significantly associated with all three outcomes. Additionally, the use of the singular pronoun “you” was robustly associated with higher TDEQ-SC (*B* = 0.26, *p* = .01) and BDI-II scores (*B* = 0.10, *p* = .05) but showed no association with SCS_pos. Furthermore, the number of words used (LIWC-num) had a positive association with BDI-II scores (*B* = 0.22, *p* < .001). No other predictor variable reached significance.

## Discussion

In the present study, we aimed to describe and compare how a sample of university students and patients with depression verbalized their inner self-critical voice (“inner critic”) by analyzing its language with LIWC-2015, and to examine whether word use is associated with self-reported trait self-criticism, self-reported self-compassion, and depressive symptoms. As evidence on the linguistic features of self-criticism is scarce and the construct is mostly assessed via self-report, we sought to explore an alternative approach to assessing self-criticism. By automatically analyzing how individuals speak to themselves, it may be possible to detect excessive self-criticism early and use this information to promote a healthier inner dialogue, thereby helping to prevent the development of psychopathological symptoms associated with excessive self-criticism [4].

Almost all relationships between the LIWC-categories and self-reported self-criticism were in the expected direction, except the number of words used by the “inner critic”. We had expected that a higher number of words used by the “inner critic” would be associated with self-reported self-criticism. However, we found no correlation between the mere number of words used by the “inner critic” and self-reported self-criticism. The number of words was, however, positively related to depressive symptoms (in both students and patients), and negatively correlated with self-compassionate responding (only in patients).

Furthermore, we expected that the clinical sample of patients with depression would overall say more words when embodying their “inner critic” compared to university students. Conversely, we found the exact opposite: university students used more words when expressing their inner critical voice than the patients. As there was no correlation between self-reported self-criticism and the number of words used by the “inner critic”, it can be concluded that the quantity of verbalized self-criticism is less relevant than the way self-criticism is expressed.

Looking at the use of the pronoun “you” (singular) by the “inner critic”, we found that it was correlated with self-reported self-criticism. This suggests that the instruction on how to express the “inner critic” was understood by the participants and that the more sentences containing “you”, the more self-criticism was verbalized (e.g., “You have not done a good job in this task. You should have concentrated more.”). The effects were of small effect size, comparable to former studies regarding word use and personality traits [45], including narcissism [53], negative emotionality [55], and self-acceptance [56], or even slightly higher. Beyond that, this observation underlines the assumption that the inner critical voice developed from internalized external criticism like parental criticism or criticism from peers or teachers [3, 17, 66], as they also would address the other person with “you”.

Regarding the use of emotion words, the results from the LIWC-analysis of verbalized self-criticism are in line with the observations of emotional states while self-criticizing [35] and add further understanding to the cognitive-affective process of state self-criticism. We found that verbally expressed self-criticism was associated with the use of fewer positive emotions in university students. Verbalizing self-criticism characterized by more negative emotion words was associated with higher trait self-criticism in patients, while there was no association among the university students. For self-compassion, we found that the “inner-critic” used fewer negative emotion words.

Additionally, we observed, only in students, a negative correlation between the use of positive emotion words and depressive symptoms. In contrast, only in the patient sample, where the “inner critic” used more negative emotion words, we found a significant negative correlation between the use of negative emotion words and depressive symptoms. However, when exploring this association with a multivariate linear regression, no significant associations were observable regarding the use of emotion words.

Overall, these observations underline the potentially health-promoting power of positive emotions [67], as it appears to be reflected in the words the “inner critic” uses. Furthermore, even though young adults are potentially at risk for high self-criticism [32], and even though the presented anagram task could have posed a higher threat to the academic self-esteem for university students compared to patients, patients expressed their inner critical voice in a more harmful manner. This may reflect that patients have a more direct access to the mental presentation of their inner critic [33].

### Limitations and implications for future research

The results of our analysis might be limited by the sample composition: potential improvements include the use of larger samples that are also matched regarding age, marital status, and educational background. Furthermore, when interpreting the results of this analysis, it is important to keep in mind the exploratory nature of this study and therefore the limited generalizability of its outcomes. Potential solutions could include replicating this work on the associations of the LIWC-categories with self-criticism, conducted with larger and more representative samples. Similarly, in the present analysis, we only considered self-criticism as assessed by the TDEQ-SC, whereas future studies could also use the *Forms of Self-criticizing/Attacking and Self-Reassuring Scale* (FSCRS) [20].

Furthermore, the experimental setting could be improved. For example, the Success Failure Manipulation using an anagram task was most likely not personally relevant for the participants, although it can be assumed that testing verbal intelligence in an academic context might be somewhat important for students. Future research should extend the experimental setting by not only inducing an experience of failure, but also applying a full success-failure paradigm to compare the activation of the “inner critic” after success [68], or by asking participants to describe a self-chosen situation in which they failed (or succeeded) and allowing them to freely talk or write about it.

Finally, a more complex multivariate statistical approach examining the interplay of self-criticism, self-compassion, and depressive symptoms could provide deeper insights into the cognitive-affective processes of state self-criticism. This is particularly relevant given the need to explore if and how the two processes, (a) hostile and self-attacking self-criticism, and (b) self-compassionate self-correction, can be distinguished. Only recently, a longitudinal study demonstrated distinct associations of self-criticism and self-compassion with mental health outcomes, indicating that these constructs are not merely two extremes of a single dimension but rather represent two separate cognitive processes that should be addressed separately in mental health interventions [69].

However, to translate the findings of this fundamental research in cognitive psychology into real-world applications, and to overcome the somewhat artificial lab setting, it may be insightful to analyze the inner critical voice in daily life, for example, using ambulatory assessment methods and examining verbalized self-criticism in response to everyday experiences of failure, with a focus on spontaneous language. Furthermore, traces of self-criticism could be detected in online forums or on social media, similar to the approach of Mesquiti and colleagues [49], by analyzing written online comments and chats.

We believe that the results, even if preliminary, have potential practical relevance for clinical practice by informing therapeutical interventions aimed at modifying cognitive-affective processes through cognitive restructuring (CBT), emotion regulation (EFT), self-compassionate self-correction (CFT), transference-countertransference work (psychodynamic psychotherapy), or emotion-focused psychodynamic psychotherapy (EFPP) [70]. To do so, counselors and therapists could learn how to identify clients and patients with a destructive inner self-critical voice based on their word use, potentially using LIWC-analysis of individuals’ written texts or transcribed audio samples, and use that as a baseline for harmful self-directed communication to guide interventions toward a more friendly and healthy inner dialogue.

Moreover, analyzing online language for early-warning signs of a “self-critical tone” could inform preventive strategies for mental health problems. This is particularly relevant for young adults and adolescents, as they are especially vulnerable to excessive self-criticism [32] compared to other age groups. Additionally, they are exposed not only to external criticism of parents, teachers, and peers but also potentially “the entire world” through their online activities (e.g., forums, social media). By detecting harmful word use early, mental health interventions could help prevent the development of mental disorders at an early stage.

## Conclusion

The results of this study contribute to current knowledge on the assessment of self-criticism, which has predominately been measured through self-report instruments. Linguistic features (word use) in verbalized self-criticism appear to reflect the general disposition toward self-criticism and are associated with depressive symptoms. Examining self-criticism in “action“ revealed novel insights into the cognitive-affective process underlying the inner critical voice, which should be further explored in future studies aimed at translating these findings into applications for mental health promotion and clinical practice.
